# Changes in ground beetle diversity and community composition in age structured forests (Coleoptera, Carabidae)

**DOI:** 10.3897/zookeys.147.2102

**Published:** 2011-11-16

**Authors:** Kathryn N. Riley, Robert A. Browne

**Affiliations:** 1 Department of Biology, Wake Forest University, Winston-Salem, NC. U.S.A.

**Keywords:** Piedmont forests, North Carolina, species richness, ecological indicators, wing-state, pitfall trap, succession, biodiversity

## Abstract

We examined diversity, community composition, and wing-state of Carabidae as a function of forest age in Piedmont North Carolina. Carabidae were collected monthly from 396 pitfall traps (12×33 sites) from March 2009 through February 2010, representing 5 forest age classes approximately 0, 10, 50, 85, and 150 years old. A total of 2,568 individuals, representing 30 genera and 63 species, were collected. Carabid species diversity, as estimated by six diversity indices, was significantly different between the oldest and youngest forest age classes for four of the six indices. Most carabid species were habitat generalists, occurring in all or most of the forest age classes. Carabid species composition varied across forest age classes. Seventeen carabid species were identified as potential candidates for ecological indicators of forest age. Non-metric multidimensional scaling (NMDS) showed separation among forest age classes in terms of carabid beetle community composition. The proportion of individuals capable of flight decreased significantly with forest age.

## Introduction

Temperate forests cover a large geographic area in the southeast United States, constituting 75.6% of total land coverage in 2003 (Chen et al. 2006). Anthropogenic activities such as farming, forestry management, and urbanization have created a patchwork of human modified land-use classes which may affect biodiversity and composition of living communities (Niemelä et al. 2000). Since it is impossible to gauge all anthropogenic impacts on the total biota, even in a small geographic area (Duelli and Obrist 1998, 2003), bioindicator taxa are often used to assess impacts of land-use change (Niemelä et al. 2000). Ideally, biodiversity indicators should reflect the diversity of other biota in a given area (McGeoch 1998; Rainio and Niemelä 2003). Ground beetles (Coleoptera: Carabidae) have been be used to indicate temperature and moisture gradients (Thiele 1977; Butterfield et al. 1995) and their community assemblages have been directly related to habitat type, ground vegetation and litter (Niemelä et al. 1993; Niemelä and Spence 1994; Koivula 2001; Rainio and Niemelä 2003). Carabidae are speciose, approximately 40,000 described species (Lövei and Sunderland 1996), and are ecologically important as generalist invertebrate predators (Larsen et al. 2002), with some species being phytophagous or having specialized feeding preferences (Thiele 1977; Lövei and Sunderland 1996).

Previous studies on the effect of forest succession or management practices indicate that Carabidae species richness and abundance increase following anthropogenic forest clear-cutting (Niemelä et al. 1993; Spence et al. 1996; Niemelä 1997; Butterfield 1997; Kiovula et al. 2002; de Warnaffe and Lebrun 2004). This has been attributed to the relatively high number of open habitat and habitat generalist carabid species with preference to the drier, open areas of grasslands and equivalent habitats (Erwin 1979; Niemelä 1993; Spence et al. 1996; Niemelä et al. 2007). Older aged forests are less diverse, with a few abundant species while the majority of species occur in low numbers (Niemelä 1993; Koivula et al. 2002). Changes in carabid assemblages along a forest age gradient indicate that some species are limited to or occur in greater numbers within specific forest age or successional stages (Niemelä et al. 1993; Larsen et al. 2003; Spence et al. 1996; Work et al. 2004; Jacobs et al. 2008). Identifying specific indicator species for forest age classes would allow for inference of habitat type or microhabitat conditions associated with forest age by the presence of particular species.

For the Carabidae, as environmental stability and time since colonization increases, the proportion of macropterous (flight enabled) individuals is predicted to decrease (Darlington 1943; Kavanaugh 1985; Liebherr 1988; Lövei and Sunderland 1996; Gutiérrez and Menéndez 1997). Since flight and the associated morphological structures (i.e. wing and muscle development) are energetically costly, there should be rapid selection against these structures if they are no longer beneficial (Darlington 1943; Kavanaugh 1985). In age structured forests, we hypothesize that the proportion of carabid species with the potential of flight will show a negative correlation with forest age.

This study quantifies species distributions of carabid beetles for five age-structured forest classes, ranging from recently cut areas (age 0) to plots with trees that are approximately 150 years old. Across the five forest classes we investigated three parameters: carabid beetle species diversity, community composition, and wing-state (as a proxy of flight ability) as well as determined species potential to as act as ecological indicators of forest age.

## Materials and Methods

**Figure 1.**
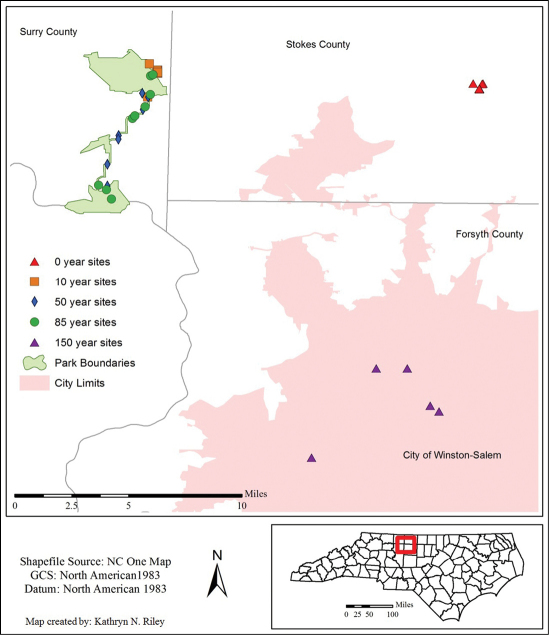
Map of the study area in Piedmont, North Carolina, with 33 sample sites indicated.

Thirty-three sites, selected to represent five forest classes, were sampled. All sites are located within Stokes, Surry, or Forsyth counties in the northern Piedmont region of North Carolina (Figure 1). Twenty two sites are located within Pilot Mountain State Park (PMSP). These sites are located in the two larger continuous sections of the park (Mountain or Yadkin River sections) or along the approximately 10 km section of the Yadkin Corridor Trail of PMSP which connects the larger sections.

The sites represent the following five approximate age categories: 0 year old forest (n = 6), 10 year old forest (n = 6), 50 year old forest (n = 7), 85 year forest (n = 9), and 150 year forest (n = 5). The 150 year old category was limited to five sites due to the difficulty of finding mature forests in the region. The uneven sampling design of the study was the result of limited site availability. Tree ages were estimated by historical records, tree girth and in selected cases via dendrological analysis. The 0 year sites are located within a 65 hectare (ha) plot which was logged in December 2008; most of the tract was clear-cut with a few mature deciduous trees left standing in riparian zones (as required by law). That plot is located approximately 23 km east of the nearest Pilot Mountain State Park site at the same altitude and classified as the same North Carolina Geological Survey (NCGS) soil type (i.e., Metagraywacke and Muscovite – Biotite Schist (CZma^2^)) (Brown 1985). The 150 year forest sites are forest fragments (< 2 ha) located within the city limits of Winston-Salem, NC. We recognize that the spatial distribution is not random. Site selection was affected by availability of forest site locations of the proper age class, urban surroundings, and permission to collect. Since the zero aged sites were located within a single, although relatively large area, we are aware of the potential for pseudoreplication among sites for this age class. However, within the zero age plot there was high heterogeneity among sites, with zero age sites located near streams, on open land and in areas of rapidly recovering vegetation.

Pitfall sampling was conducted monthly for one year, from March 2009 through February 2010. Pitfall trapping has been shown to be appropriate for studies comparing species richness and activity/abundance levels of larger (> 5 mm) ground-dwelling beetles (Adis 1979; Thiele 1977; Butterfield 1997; Dufrêne and Legendre 1997; Werner and Raffa 2000; de Warnaffe 2004, etc.). Although pitfall trapping has been criticized for its dependence on weather, beetle activity, and selection for larger individuals (Greenslade 1964; Thiele 1977; Adis 1979; Lövei and Sunderland 1996; Liebherr and Mahar 1979; Werner and Raffa 2000; Larsen et al. 2003), it has shown to be a useful collection technique for ecological studies. The passive sampling approach of pitfall trapping minimizes the investigator associated biases inherent in active search method. Within the goals of this study, we believe that pitfall traps are an efficient, valuable means of collection (Thiele 1977; Butterfield 1997; Larsen et al. 2000). Pitfall traps consisted of a plastic drinking cup (Solo brand, with a 12 cm diameter at the mouth), positioned flush with the ground. A dense foam plastic cover was loosely placed over each trap and fixed to the ground using nails, in order to keep rain and other debris out of the trap. Each cup contained antifreeze as a preservative. Each study site was divided into two groups of six cups (6 A and 6 B) located approximately 10 m apart for a total of 12 traps per site. Carabidae were collected from each trap monthly throughout the year-long study by straining the trap contents through a fine mesh screen and then preserved in 95% ethanol. Raw count of individuals collected each month were adjusted to reflect number of individuals collected for 12 pitfall traps per 30 day period. This corrected for variations in collection times and pitfall trap disturbance (i.e. overturned) at each site. To correct for large variances within and among study sites and to adjust for potential non-normal distribution, the mean abundance for each forest age classification was natural log transformed. Individuals were identified to the species level using a regional morphological key (Ciegler 2000)and by comparingselected individuals with specimens housed at the Smithsonian National Museum of Natural History.

### Data analysis

Six indices were used to directly or indirectly calculate diversity of carabid beetles for each forest age class: species richness (S), Shannon diversity index (H’) (Shannon and Weaver 1949), Shannon’s evenness (J), Simpson’s diversity index (1-D) (Simpson 1949), Fisher’s alpha (Fisher et al. 1943), and Berger-Parker dominance (d) (Berger and Parker 1970). The mean and 95% confidence interval of all diversity indices were calculated for each forest age class. Completely Randomized Design ANOVA and Tukey’s test for multiple comparisons were primarily used to determine if the means of five of the diversity measures were statistically different among forest age classes (Hampton and Havel 2006). Due to uncorrectable heteroscedasticity for Simpson’s values, a Games and Howell test for unplanned comparison of means (Games and Howell 1976; Sokal and Rohlf 1995) replaced ANOVA tests. Species accumulation curves (Colewell 1997) were computed using EstimateS (v8.2.0, 2009) with corrected abundance data for each site and subjected to 500 randomizations. Species number (referred to as S_obs_ Mao Tau) was plotted against the number of individuals for each of the five age classes. This technique calculates the rate at which species are being accumulated as sampling effort increases i.e., individuals collected (Smith and Smith 2006). To compare differences in the rate of species accumulation among forest age classes, a χ^2^ goodness-of-fit test was performed, with a null hypothesis that the expected number of individuals at each forest age class would be random. The point on the sampling curve chosen to conduct the test was n = 233 (the lowest number of individuals collected from any of the five forest ages).

Species were classified in terms of rarity based on the proportion of their abundance to the overall carabid beetle catch for the sampling period (i.e. one trapping season). Rarity categories, which were designated according to the percent of the total sample collected, are as follows: singleton (one individual, 0–0.05%), doubleton (two individuals, 0.06%-–0.10%), extremely rare (0.11%–0.20%), rare (0.21%–0.99%), common (1.00%–9.99%), and dominant (10.00%–19.99%).Chi-square goodness of fit tests were calculated for the total catch for each of the 5 age classes to detect deviations from expected distributions.

Non-metric multidimensional scaling (NMDS) was utilized (PC-Ord, Version 5, McCune et al. 2002) to examine community composition of carabids for the five forest age classes. NMDS ordination uses Sørensen’s similarity, also known as the Bray-Curtis coefficient, to measure the dissimilarity in species compositions among forest age classes. Since rarer species may negatively affect the ordination results (Dufrêne and Legendre 1997; Palmer 2009) NMDS analysis was conducted using 17 species classified as common or dominant according to the rarity scale of this study (Table 2).

**Table 1.** Number of replicates, carabid beetle abundance, and the raw number of genera and species for each forest age class.

Site	Number of Replicates	Abundance	Avg. Abundance	Genera	Species
0	6	443.26	73.88	22	43
10	6	538.85	89.81	21	33
50	7	626.72	89.53	19	30
85	9	725.61	80.62	22	34
150	5	233.06	46.61	11	17
Total	33	2567.51	77.80	30	63

### Wing-state

Carabid species were classified into one of two dichotomous states: macropterous or brachypterous. Individuals were considered macropterous when fully developed wings combined with the potential for flight were documented, while brachypterous beetles include beetles with short wings (brachyptery) with low potential for flight and beetles lacking wings (aptery) with virtually no flight potential. Wing-state for each species was determined by visually examining the degree of hind wing development of at least five individuals (for species with n ≥ 5) as well as consulting the degree of flight capability cited in the literature (reviewed by Larochelle and Larivière 2003).

### Ecological indicators

To identify species as potential candidates for indicators of forest age, each species was classified according to the total number of individuals collected from each of the five forest age classes. Species with n < 5 individuals were excluded from subsequent analysis. Species were classified as follows: extreme habitat generalist (occurred in all 5 forest age classes), habitat generalist (occurred in 4 forest age classes), habitat intermediate (occurred in 3 forest age classes), habitat specialist (occurred in 2 forest age classes), and extreme habitat specialist (occurred in 1 forest age class).

Species classified as a habitat specialist, extreme habitat specialist, or species exhibiting a peak in abundance for any stage of the forest age gradient, were examined as potential candidates for ecological indicators of forest age. An additional requirement for candidacy was the presence of the species (minimum of one individual) in at least 50% of the replicates within the particular forest age class. A χ^2^ goodness of fit test was then used to determine whether the distribution of species abundance among the forest age classes was due to chance alone. In order to meet the requirement of a χ^2^ test (with a minimum expected value of n = 5 for each class), only species where n ≥ 25 individuals were tested. If species distribution among classes was statistically significant, the null hypothesis was rejected and we concluded that distribution did not occur due to chance alone. Subsequently, an additional clumped chi-square analysis was conducted to determine if the forest age class(es) with the highest abundance contained more individuals than expected compared to all other forest classes combined (see Riley 2010).

**Table 2.** Carabidae species (S = 63) with corrected abundance for forest age class. Macropterous (M) species have the potential for flight and brachypterous (B) species are considered to be flightless. Abbreviations for specialty habitat categories for 35 species (n > 5) are: EG = extreme generalist, G = generalist, I = intermediate, S = specialist, ES = extreme specialist. See text for description of each category. Species with an asterisk (*) are the 17 species most commonly collected in the study.

Species	Wing State	Specialty Category	0	10	50	85	150	Total
*Agonum punctiforme* (Fabricius)	M		4.83					4.83
*Amara aenea* (De Geer)	M	S	22.98		1.05			24.02
*Amara crassispina* (LeConte)	M	S	5.58	1.52				7.10
*Amara cupreolata* (Putzeys)	M		2.21	0.99	1.17			4.37
Amara familiaris* (Duftschmid)	M	I	19.57		14.25	0.95		34.77
*Amara impuncticollis* (Say)	M	S	4.29		4.81			9.10
*Amara musculis* (Say)	M		1.04					1.04
*Amphasia interstitialis* (Say)	M			1.90		1.22		3.12
*Anisodactylus carbonarius* (Say)	M		1.13					1.13
*Anisodactylus furvus* (LeConte)	M		2.33					2.33
*Anisodactylus haplomus* (Chaudior)	M		0.92					0.92
*Anisodactylus harrisii* (LeConte)	M	S	8.15	1.26				9.41
*Anisodactylus rusticus* (Say)	M	ES	7.79					7.79
*Apenes lucidulus* (Dejean)	M			1.14	0.84	0.84		2.83
Calathus opaculus* (LeConte)	M	G	4.57	31.66	84.05	14.49		134.77
Carabus goryi* (Dejean)	B	G	17.44	0.82	5.37	14.41		38.05
*Carabus sylvosus* (Say)	B	I	4.82	0.92	1.94			7.68
Chlaenius aestivus* (Say)	B	EG	21.13	19.00	2.23	17.86	4.83	65.06
*Chlaenius amoenus* (Dejean)	M	G	1.84	0.95		1.94	12.12	16.85
*Chlaenius emarginatus* (Say)	M	EG		6.81				6.81
*Cicindela sexguttata* (Fabricius)	M	I		2.80	2.43	3.00		8.98
*Cicindela unipunctata* (Fabricius)	M					0.92		0.92
*Colliuris pensylvanica* (Linnaeus)	M		1.11					1.11
Cyclotrachelus freitagi* (Bousquet)	M	EG	0.98	6.40	41.58	14.02	0.77	63.75
Cyclotrachelus sigillatus* (Say)	B	EG	98.46	23.96	75.30	155.88	104.92	458.52
Cyclotrachelus vinctus* (LeConte)	B	EG	0.87	171.90	5.78	26.83	6.20	211.57
*Cymindis americanus* (Dejean)	B	EG	0.92	2.89	1.01	3.77	1.01	9.61
*Cymindis limbatus* (Dejean)	M			1.84				1.84
*Cymindis neglectus* (Haldeman)	B					1.22		1.22
*Cymindis platicollis* (Say)	M					2.34		2.34
Dicaelus ambiguus* (LaFerté-Sénecteré)	B	EG	2.03	24.74	11.80	19.13	8.52	66.22
Dicaelus dilatatus* (Say)	B	G	3.17	11.45	23.87	4.86		43.35
*Dicaelus elongatus* (Bonelli)	B	I	5.71	2.01	4.40			12.12
Dicaelus politus* (Dejean)	B	EG	0.92	10.93	1.72	12.87	11.59	38.02
*Dicaelus purpuratus* (Bonelli)	B					0.84		0.84
Galerita bicolor* (Dry)	M	EG	2.31	20.54	6.25	58.18	6.33	93.60
*Harpalus compar* (Leconte)	M		1.13					1.13
*Harpalus erythropus* (Dejean)	M				0.82			0.82
*Harpalus fulgens* (Csiki)	B		1.35					1.35
Harpalus herbivagus* (Say)	M	ES	36.41					36.41
*Harpalus katiae* (Battoni)	M		1.13					1.13
Harpalus pensylvanica* (De Geer)	M	EG	58.72	2.90	22.42	4.90	1.50	90.43
*Harpalus spadiceus* (Dejean)	B					1.90		1.90
*Megacephala virginica* (Linnaeus)	M		1.13	0.92				2.05
*Myas coracinus* (Say)	B	S	12.77				2.84	15.61
*Notiobia terminata* (Say)	M		0.92					0.92
*Notiophilus aeneus* (Herbst)	M				1.22	0.78		2.00
*Oodes fluvialis* (LeConte)	M				1.22			1.22
Pasimachus punctulatus* (Haldeman)	B	G	23.25	5.70	0.95	2.69		32.59
*Patrobus longicornis* (Say)	B						0.53	0.53
*Platynus decentis* (Say)	B	I	2.05		9.39	7.69		19.13
*Poecilus lucublandus* (Bonelli)	M	EG	3.99	0.95	6.38	2.30	0.77	14.39
Pterostichus coracinus* (Newman)	B	EG	11.34	4.97	3.67	34.14	6.94	61.06
*Pterostichus moestus* (Say)	B	ES				9.60		9.60
Pterostichus sculptus* (LeConte)	B	EG	22.99	34.83	228.96	71.65	1.27	359.70
*Rhadine caudata* (LeConte)	B	S		1.97		3.66		5.63
*Scaphinotus andrewsii* (Valentine)	B	I		4.74	1.17	5.06		10.96
*Scaphinotus unicolor* (Fabricius)	B		1.90					1.90
*Scaphinotus violaceus* (LeConte)	B			0.95				0.95
*Scarites subterraneus* (Fabricius)	M	I	4.85	4.40		1.22		10.46
*Selenophorus ellipticus* (Dejean)	M					1.17		1.17
*Selenophorus opalinus* (LeConte)	M						0.84	0.84
Sphaeroderus stenostomus* (Weber)	B	EG	12.23	130.08	60.67	222.53	62.09	487.60
Total			443.26	538.85	626.72	725.61	233.06	2567.51

## Results

### Abundance

A total of 2,568 individual ground beetles representing 30 genera and 63 species were collected from 33 study sites (Table 1). Adjusted carabid beetle abundances relative to the five forest age groups are listed in Table 2. No significant differences occurred for average carabid abundance among the 5 forest age classes. Carabid beetle abundance was highest in the 10 and 50 year forest classes and lowest in the 150 year old forest (Table 1).

### Diversity

For most measures of carabid diversity the 0 age forest class had the highest values while the lowest values, except for dominance, were found for the 150 year forest class (Table 3). Species richness varied significantly between the 0 and 150 year old forest classes (ANOVA, with Tukey test, p < 0.002). Shannon diversity (p < 0.005) and Simpson diversity (Games and Howell test, p = 0.05) show similar trends between the 0 and the 150 aged forests. Dominance peaked for 150 year old forest class (0.55), with the lowest value (0.35) for the 10 year old forest class. Forest age is negatively correlated with species richness (r = -0.945, p = 0.016), Shannon diversity (r= -0.916, p = 0.029), and Simpson’s diversity (r = -0.926, p = 0.024).

**Figure 2.**
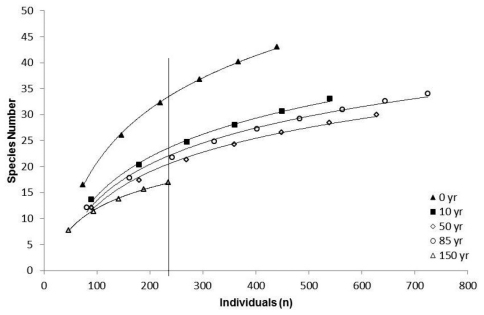
Carabid beetle species accumulation curves for five forest age classes. Vertical line indicates species richness of each curve at n = 233 individuals.

Species accumulation curves for all forest age classes did not reach an asymptote (Figure 2), indicating that additional sampling effort would likely uncover uncollected species (Smith and Smith 2006). No significant difference (χ^2 ^= 6.50, 4 df, p = 0.05) was found in number of species among the five forest age classes at the point in the curves where n = 235 (which represents the number of individuals sampled from the forest age class with the lowest overall abundance).

**Table 3.** Values for diversity indices for five forest age classes.

Age	S	95% CI	H’	95% CI	Simpson(1-D)	95% CI	Fisher’s Alpha	95% CI	d	95% CI
0	16.50	1.87	2.12	0.19	0.81	0.06	11.80	1.09	0.36	0.11
10	13.67	4.13	1.83	0.17	0.78	0.03	7.75	0.74	0.35	0.07
50	12.14	3.67	1.78	0.34	0.75	0.12	6.56	0.55	0.47	0.17
85	12.11	2.57	1.79	0.25	0.76	0.08	7.4	0.47	0.39	0.10
150	7.80	2.77	1.33	0.45	0.63	0.21	4.21	0.68	0.55	0.23

### Community structure

Most species (47 out of 63) occurred in low abundance and were classified in the three rarest categories in the analysis. The highest number of singletons (S = 8) and rare species (S = 15) occurred in the recently logged forest. However, the uneven number of age replicates should be taken into consideration when interpreting differences in rarity among age classes. For example, the 150 year forest class contained the lowest number of replicates, and also had the lowest value for total species richness. Three species, *Cyclotrachelus sigillatus* (Say, 1823), *Pterostichus sculptus* (LeConte, 1852), and *Sphaeroderus stenostomus* (Weber, 1801), occurred in all forest classes, constituting 51% of the total number of individuals collected in the study.

**Figure 3.**
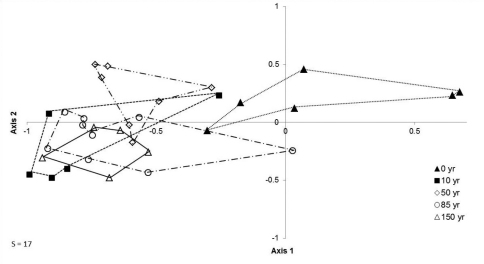
Results of Non-Metric Multidimensional Scaling (NMDS) analysis for 33 study sites. The analysis was based on the 17 most common carabid beetle species from five forest age classes (0, 10, 50, 85, and 150 years). Each the five polygons represent different forest age classes, as indicated by different symbols.

NMDS indicates a grouping of age replicates, with at least partial separation of sites by age for all but one of the forest classes (Figure 3), suggesting similarity in carabid species assemblages for equivalent forest classes. The two axes in NMDS ordination accounted for 63% of the variance in species composition (r^2^: axis 1 = .447, axis 2 = .185) for the 17 most commonly occurring species among the 33 study sites. The 0 and 50 age year polygons have the highest scores on axis 2 and exhibit greater separation from the 10, 85, and 150 year old polygons. The polygons demonstrate a convergence in ordination space as forest age increases, indicating that variability in species composition decreases as forests age.

### Wing-state

**Figure 4.**
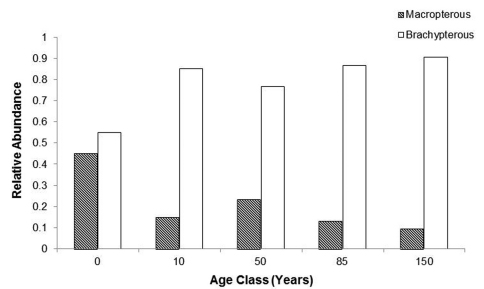
Proportions of carabid populations brachypterous and macropterous for five forest age classes. Significant differences occurred for all forest age classes except the zero age class (χ^2^ < 0.05).

The majority of the species collected (39 of 63) were fully winged (macropterous) (Table 2). The remaining species were brachypterous (includes specimens with fused elytra) and are deemed incapable of flight. The difference in macropterous versus brachypterous individuals within each forest age group was significant except for 0 age forest (χ^2 ^< 0.05). The number of macropterous individuals decreases as forest age increases (Figure 4) although the correlation between macroptery and forest age was not significant.

### Ecological indicators

**Figure 5.**
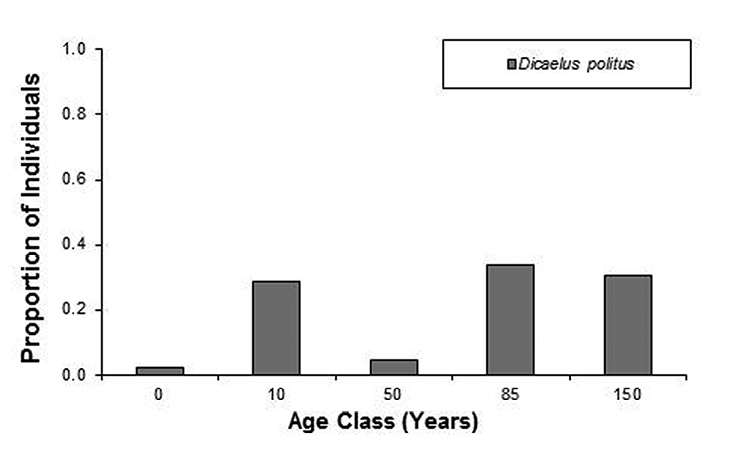
Relative abundance across the forest age gradient for a representative species from each of the indicator classes (see text for definitions of indicator classes).

For the 35 species where n > 5 (see Table 2), 14 were classified as extreme habitat generalists (i.e. collected within all forest age classes). Distribution of species among the five habitat specialty categories is not random, with more species occurring in the generalist categories (χ^2^ < 0.05). Of the 25 species where n > 10, 17 species were identified as having strong affinities for forest age classes (χ^2^ < 0.05), with 15 species showing preference for a single forest age class and 2 species preferring contiguous forest age classes (see Figure 5 for representative species of each indicator class).

## Discussion

### Abundance

The intermediate forest age classes (10, 50, and 85 year old forests) yielded the largest number of individuals, while the two extreme age classes (0 and 150 year old forests) had lower levels of abundance. Although these results depict a trend and are not statistically significant, they provide support for previous studies which reported higher carabid abundance in intermediate aged boreal forest classes (Spence et al. 1996; Koivula et al. 2002). Several investigations have reported lower carabid activity-abundance in clear-cut sites compared to more mature forest classes including younger regeneration classes (5-15 years) (Jennings et al. 1986; Niemelä et al. 1993; Koivula et al. 2002).

Results from this study of deciduous Piedmont North Carolina forest sites follow a similar pattern following a clear-cutting event similar to that found for carabid communities of northern European and high latitude boreal forests (Lenski 1982; Niemelä et al. 1993; Butterfield et al. 1995; Spence et al. 1996; Niemelä 1997; Butterfield 1997; Koivula 2001; Koivula et al. 2002; de Warnaffe and Lebrun 2004). Open habitat species were reported to occur at high densities for the first 20-30 years following clear-cutting until the forest canopy closes (Niemelä et al. 1993, 1996; Butterfield 1997; Koivula et al. 2002). There are several possible reasons for this observed high species richness in younger forests. A high proportion of grassland and meadow carabid species prefer warmer, open habitats such as those found in recently disturbed forests (Erwin 1979; Niemelä 1993; Niemelä et al. 2007). Since many open habitat carabid species consume seeds, the presence of this type of food and its abundance may strongly influence whether they are present in clear-cut areas (Erwin 1979). In Iowa, the highest carabid beetle diversity and abundance were reported from tall-grass prairie habitats, with the second highest values found in wooded forests (Larsen et al. 2003). Following a severe disturbance of mature forests (as occurs with clear-cutting) there is an influx of rapidly colonizing, open habitat carabid species in combination with the temporary presence of the carabid generalists more typical of mature forests. Our results indicate that the temporary increased diversity of carabid species in recently clear-cut forest sites is due to the influx of open habitat carabid species. In contrast, there is a small amount of evidence for the increase of carabid species diversity due to the persistence of mature forest species in the clear-cut forest sites.

### Community structure

Most species collected in this study occurred as singletons or were classified as rare. Carabid beetle assemblages from mature forests are often dominated by a few abundant species, while the majority of species are either scarce or occur at low abundance levels (Niemelä 1993; Koivula et al. 2002). Despite having a relatively low number of collected individuals, the 0 age forest class contained the highest number of singletons and rare species. Young forests, with a high flux of individuals, many with good dispersing abilities, are environmentally less stable for carabid beetles than mature forests (Butterfield 1997; Gutiérrez and Menéndez 1997). In this study, high numbers of rapidly colonizing species probably entered the open areas following clear-cutting and will persist only for short periods, resulting in high species turnover for the 0 aged forest sites. Low abundance may be attributed to the inability of open habitat species to establish long term communities at these sites due to unstable conditions. In addition, highly dispersing carabid species may not be as susceptible to capture through pitfall trapping and therefore would not be collected as often or at all.

Species richness can be a misleading indicator of conservation value because disturbed sites, while high in species diversity, will often be characterized by widespread, abundant generalist species (Spence et al. 1996; Koivula et al. 2002; Niemelä et al 2007; Paquin 2008). As Lenski (1982) has noted, a clear-cut event disrupts the competitive exclusion of more stable, mature forest habitats, allowing for the increase of diversity of carabid species, although most species are within the same genus and/or share similar life characteristics.

NMDS generated age polygons for each forest class, with the exception of 10 year sites, occur sequentially by forest age when moving negatively in the two dimensional space depicted. Most of the 10 year sites are “out of sequence”, i.e. they overlap or are located in proximity to the 85 and 150 year sites. This suggests that the 10 year sites are more similar in carabid species composition to more mature forested sites. In the experimental design of our field collections, most of the 10 year replicates are located adjacent to 85 year sites. This proximity creates the potential for species commonly found in more mature forests to immigrate into younger forest stands (Koivula et al. 2002). It is possible that the ten year sites were not isolated enough to provide true insight to a forest of this younger age class. The NMDS ordination analysis suggests that 0 and 50 year site polygons differ the most from the other age sites in terms of species composition. NMDS ordination analysis also indicates that the distance between the age polygons (with the exception of the 10 year group), becomes shorter as forest age increases, suggesting that the carabid communities of younger forests change relatively rapidly and that carabid community stability increases with forest age (Butterfield et al. 1995).

NMDS ordination analysis indicates that distinct communities of ground beetles occur along the forest age gradient, especially among younger aged forest classes. Forest canopy closure is a time of drastic change in carabid community assemblage (Niemelä et al. 1993, 1996, Butterfield et al. 1995, Butterfield 1997, Paquin 2008). In Piedmont North Carolina rapidly growing pines are able to form a closed canopy above other seedlings approximately 5-15 years post-abandonment. At 70-100 years the amount of light sufficiently decreases, leading to pine mortality, and young hardwoods are able to fill the canopy gaps (Peet and Christensen 1980, 1987; Christensen and Peet 1984). Both canopy closure and the change in dominance tree species from pines to hardwoods may influence the carabid communities. One of the major associated changes is the quantity of sunlight reaching the forest floor which influences temperature and humidity (Butterfield et al. 1995; Jacobs et al. 2008). Microclimatic conditions including moisture, temperature, and understory structure have been reported as potential factors influencing notable changes in carabid beetle communities (Niemelä et al. 1996; Butterfield 1997).

### Wing-state

Differences in the proportions of macropterous versus brachypterous individuals among forest age classes are striking, with significantly more brachypterous than macropterous individuals for all forest age classes, except for 0 year forest class. As forest age increases the number of macropterous carabid beetles decreases, suggesting that the advantage of flight decreases with forest maturity. In stable environments, dispersal would be less important for carabid beetle survival and reproduction, allowing resources that would otherwise be used for wing development to be reallocated to developmental and/or reproductive needs (Darlington 1943; Thiele 1977; Kavanaugh 1985; Liebherr 1988; Gutiérrez and Menéndez 1997). The highest proportions of macropterous individuals were collected at the 0 forest age class, where the trees were cut only three months prior to the start of this study. The rapid colonization of open-habitat species to the disturbed area reflects the high dispersal ability of these species (Niemelä et al. 1996).

### Ecological indicators

Previous work with Carabidae suggests that the best approach to understanding the factors shaping presence and abundance is through individual species responses (Niemelä et al. 2007) rather than more general indicators such as raw species counts. The use of carabids as environmental and ecological indicators has been supported by several studies (Niemelä et al. 1993, 2000; Niemelä 1999; Larsen et al. 2003; Rainio and Niemelä 2003; Work et al. 2008). Criticisms for ground beetles as bioindicators were taken into consideration for the experimental design of this study. High spatial replication within each age class and collections over an entire year, not only during the active season, help address the issues of patchy distribution and low abundance of particular species (Rainio and Niemelä 2003). Although all of the 0 year sites are more closely located in geographical space than those for other age classes, the high heterogeneity in species composition among the replicates suggests they are separate entities at least with regards in terms of Carabidae. Our results indicate that specific carabid species are associated with different aged forests, while other carabid species are generalists, occurring in all forest ages classes. In total, 17 species exhibit a differential response to variations in forest age classes. There are seven species that could potentially serve as indicators of 0 aged forests: *Amara aenea*, *Amara familaris*, *Carabus goryi*, *Harpalus herbivagus*, *Harpalus pensylvanica*, *Myas coracinus*, and *Pasimachus punctulatus*. Two species could potentially serve as indicators for 10 year old forests: *Cyclotrachelus vinctus* and *Dicaelus ambiguus*. For 50 year old forests there were four potential indicators species: *Calathus opaculus*, *Cyclotrachelus freitagi*, *Dicaelus dilatatus*, *Pterostichus sculptus*, while 85 year old forest had two indicator species: *Galertia bicolor* and *Pterostichus coracinus*. *Platynus decentis* was found to be an indicator for 50 and 85 year old forests but did not occur in high abundance in the oldest age class while *Dicaelus politus* was statistically more abundant in the two oldest forest age classes. This relatively high number of indicator species supports the use of Carabidae as ecological indicators of age structure in deciduous southeastern U.S. forests.

Six species analyzed for habitat specialty were classified as habitat specialists (occurring in two forest age classes): *Amara aenea*. *Amara crassispina*, *Amara impuncticollis*, *Anisodactylus harrisii*, *Myas coracinus*, *and Rhadine caudata*. Additionally, three species were found to be extreme specialists (occurring in one forest age class): *Harpalus herbivagus*, *Pterostichus moestus*, and *Anisodactylus rusticus*. Most species designated as possible ecological indicators in this study were considered extreme generalists or generalists. There was a greater number of indicator species for young, open forest habitats compared to more mature forests. However, this may be an artifact of the larger number of species found in younger forests. Of the seven species showing an affinity for conditions of a recently logged forest (0 forest age class) two species were in the genus *Amara* and two species in the genus *Harpalus*; both genera are known to contain seed-eating and phytophagous species which prefer dry, open, and grassy habitats (Thiele 1977; Erwin 1979, 1981; Niemelä et al. 1993, 2007; Butterfield 1997). *Myas coracinus* (Say, 1823) and *Carabus goryi* (Dejean, 1831) were also classified as indicators of open habitats. This observation is not in agreement with previous studies where these species are stated to prefer the shaded and moist ground conditions provided by more mature forests (Larochelle and Larivière 2003; Larsen et al. 2003). It’s possible these species generally occur in more mature forests but individuals still remained in the cleared forest since the study began only three months after logging as suggested by Jacobs et al. (2008).

No carabids in this study were found as indicator species for the most mature forest age group. However, the 150 year sites are all smaller forest fragments located within the city limits of Winston-Salem, NC. The small patch size, as well as the urban surroundings, could possibly be responsible for the low overall carabid abundance and diversity for this age group.

Minimal difference in species composition occurred between the 85 and 150 year sites. Although the 150 year old forests are small patches in an urban setting their composition is similar to the 85 year sites. However, Niemelä et al. (2002) detected differences in carabid species composition, richness, and other diversity indices along a urban-rural disturbance gradient. Lack of specialists in the more mature forests may indicate these secondary forests are already too disturbed and/or fragmented, with stenotypic species never regaining sufficient populations (Rainio and Niemelä 2003). Alternatively, a small number of specialists might be expected for any similarly aged forest habitat (including less fragmented, non-urban sites) since less than 10% of predatory arthropods have been described as old growth specialists (Niemelä 1999). Differences in species occurrence across the forest regeneration cycle reinforce the need to conserve a range of age classes at the landscape level (Niemelä et al. 1996, 2007).

The role of the natural forest regeneration cycle on the diversity and composition of carabid beetles after a cutting event has been more intensely studied in northern boreal forests (Butterfield et al. 1995; Spence et al. 1996; Butterfield 1997; Niemelä et al. 1993, 1996, 2007; Jacobs et al. 2008) than in the temperate forests represented in this study. The Carabidae in this study exhibited the same general response to a logging event in terms of species richness to those previously reported for boreal forests. Additionally, there were profound changes in community composition. Additional work is needed, not only in the temperate deciduous forests, but also in additional forest types (e.g., tropical lowland forests, montane tropical forests) before carabid responses to forest disturbance can be categorized at both the individual species and community levels.

## References

[B1] Adis J (1979) Problems of interpreting arthropod sampling with pitfall traps. Zoologischer Anzeiger 202: 177-184.

[B2] Berger WH Berger FL (1970) Diversity of planktonic foraminifera in deep-sea sediments. 168: 1345-1347. 10.1126/science.168.3937.1345 17731043

[B3] Butterfield J Luff LM Baines M Eyre MD (1995) Carabid beetle communities as indicators of conservation potential in upland forests. Forest Ecology and Management 79: 63-77. 10.1016/0378-1127(95)03620-2

[B4] Chen H Tian H Liu M Melillo J Pan S Zhang C (2006) Effect of land-cover change in terrestrial carbon dynamics in the southern United States. Journal of Environmental Quality 35: 1533-1547. 10.2134/jeq2005.0198 16825474

[B5] Christensen NL Peet RK (1984) Convergence during secondary forest succession. Journal of Ecology 72: 25-36. 10.2307/2260004

[B6] Ciegler JC (2000) Ground Beetles and Wrinkled Bark Beetles of South Carolina (Coleoptera: Geadephaga: Carabidae and Rhysodidae) Clemson University, Clemson, SC, 149 pp.

[B7] Colwell R (2009) EstimateS: Statistical estimation of species richness and shared species from samples. Version 8.2. [User’s Guide and application published at: http://purl.oclc.org/estimates]

[B8] Darlington PJ (1943) Carabidae of mountains and islands: data on the evolution of isolated faunas, and on atrophy of wings. Ecological Monographs 13: 37-61. 10.2307/1943589

[B9] de Warnaffe GB Lebrun P (2004) Effects of forest management on carabid beetles in Belgium: implications for biodiversity conservation. Biological Conservation 118: 219-234. 10.1016/j.biocon.2003.08.015

[B10] Dufrêne M Legendre P (1997) Species assemblages and indicator species: the need for a flexible asymmetrical approach. Ecological Monographs 67: 345-366.

[B11] Duelli P Obrist MK (1998) In search of the best correlates for local organismal biodiversity in cultivated areas. Biodiversity and Conservation 7: 297-309. 10.1023/A:1008873510817

[B12] Duelli P Obrist MK (2003) Biodiversity indicators: the choice of values and measures. Agriculture, Ecosystems and Environment 98: 87-98. 10.1016/S0167-8809(03)00072-0

[B13] Erwin TL (1979) Thoughts on the evolutionary history of ground beetles: hypotheses generated from comparative faunal analyses of lowland forest sites in temperate and tropical regions. In: Erwin TL Ball GE Whitehead DR Halpern AL (Eds). Carabid Beetles: Their Evolution, Natural History, and Classification. Dr W. Junk bv. Publishers, The Hague: 539-592.

[B14] Erwin TL (1981) Natural history of Plummer’s Island, Maryland. XXVI. The ground beetles of a temperate forest site (Coleoptera: Carabidae): an analysis of fauna in relation to size, habitat selection, vagility, seasonality, and extinction. Bulletin of the Biological Society of Washington 5: 105-224.

[B15] Fisher RA Corbet AS Williams CB (1943) The relation between the number of species and the number of individuals in a random sample of an animal population. Journal of Animal Ecology 12: 42-58. 10.2307/1411

[B16] Games PA Howell JF (1976) Pairwise multiple comparison procedures with unequal N’s and/or variances: a Monte Carlo study. Journal of Educational Statistics 1: 113-125. 10.2307/1164979

[B17] Greenslade PJM (1964) Pitfall trapping as a method for studying populations of Carabidae (Coleoptera). Journal of Animal Ecology 33: 301-310. 10.2307/2632

[B18] Gutiérrez D Menéndez R (1997) Patterns in the distribution, abundance, and body size of carabid beetles (Coleoptera: Carabidae) in relation to dispersal ability. Journal of Biogeography 24: 903-914.

[B19] Hampton RE Havel JE (2006) Introductory Biological Statistics 2^nd^ ed. Waveland Press, Inc., 169 pp.

[B20] Jacobs J Work TT Spence JR (2008) Influences of succession and harvest intensity on ground beetle (Coleoptera:Carabidae) populations in the boreal mixed-wood forests of Alberta, Canada: species mater. In: Penev L Erwin TL Assmann T, (Eds). Back to the Roots and Back to the Future? Towards a New Synthesis between Taxonomic, Ecological, and Biogeographical Approaches in Carabidology. Proceedings of the XIII European Carabidologists Meeting. (Blagoevrgard (Bulgaria), August 2007, Pensoft, Sophia, Bulgaria and Moscow, Russia: 425-450.

[B21] Jennings DT Houseweart MW Dunn GA (1986) Carabid beetles (Coleoptera: Carabidae) associated with strip clearcut and dense spruce-fir forests of Maine. Coleopterists Bulletin 40: 251-263.

[B22] Kavanaugh DH (1985) On wing atrophy in carabid beetles (Coleoptera: Carabidae), with special reference to Nearctic *Nebria*. In: Ball GE (Ed). Taxonomy, Phylogeny, and Zoogeography of Beetles and Ants. Dr. W. Junk Publishers, The Hague: 371-372.

[B23] Koivula M (2001) Carabid beetles (Coleoptera: Carabidae) in boreal managed forests – meso-scale ecological patterns in relation to modern forestry. PhD thesis, Helsinki, Finland: University of Helsinki.

[B24] Koivula M Kukkonen J Niemelä J (2002) Boreal carabid beetle (Coleoptera, Carabidae) assemblages along the clear-cut originated succession gradient. Biodiversity and Conservation 11: 1269-1288. 10.1023/A:1016018702894

[B26] Larochelle A Larivière M-C (2003) A natural history of the ground-beetles (Coleoptera: Carabidae) of America north of Mexico. Pensoft Publishers, Sofia, Bulgaria, 583 pp.

[B27] Larsen KJ Work TT Purrington FF (2003) Habitat use patterns by ground beetles (Coleoptera: Carabidae) of northeastern Iowa. Pedobologia 11: 288-299. 10.1078/0031-4056-00192

[B28] Lenski RE (1982) The impact of forest cutting on the diversity of ground beetles (Coleoptera: Carabidae) in the Southern Appalachians. Ecological Entomology: 385–390. 10.1111/j.1365-2311.1982.tb00680.x

[B29] Liebherr J (1988) Gene flow in ground beetles (Coleoptera: Carabidae) of differing habitat preference and flight-wing development. Evolution 42: 129-137. 10.2307/2409121 28563855

[B30] Liebherr J Mahar J (1979) The carabid fauna of the upland oak forest in Michigan: survey and analysis. Coleopterists Bulletin 33: 183-197.

[B31] Lövei GL Sunderland KD (1996) Ecology and behavior of ground beetles (Coleoptera: Carabidae). Annual Review of Entomology 41: 231-256. 10.1146/annurev.en.41.010196.001311 15012329

[B32] McCune B Grace JB Urban DL (2002) Analysis of ecological communities. MjM Software Design, 304 pp.

[B33] McGeoch MA (1998) The selection, testing, and application of terrestrial insects as bioindicators. Biological Review 73: 181-201. 10.1017/S000632319700515X

[B34] Niemelä JK (1993) Mystery of the missing species: species-abundance distribution of boreal ground-beetles. Annules Zoologici Fennici 30: 169-172.

[B35] Niemelä JK (1997) Invertebrates and boreal forest management. Conservation Biology 11: 601-610. 10.1046/j.1523-1739.1997.06008.x

[B36] Niemelä JK (1999) Management in relation to disturbance in the boreal forest. Forest Ecology and Management 115: 127-134. 10.1016/S0378-1127(98)00393-4

[B37] Niemelä JK Spence JR (1994) Distribution of forest dwelling carabids (Coleoptera): spatial scale and the concept of communities. Ecography 17: 166-175. 10.1111/j.1600-0587.1994.tb00090.x

[B38] Niemelä JK Langor D Spence JR (1993) Effects of clear-cut harvesting on boreal ground-beetle (Coleoptera: Carabidae) in western Canada. Conservation Biology 7: 551-561. 10.1046/j.1523-1739.1993.07030551.x

[B39] Niemelä JK Haila Y Punttila P (1996) The importance of small-scale heterogeneity in boreal forests: variation in diversity of forest floor invertebrates across the succession gradient. Ecography 19: 352-368.

[B40] Niemelä JK Koivula M Kotze DJ (2007) The effects of forestry on carabid beetles (Coleoptera: Carabidae) in boreal forests. Journal of Insect Conservation 11: 5-18. 10.1007/s10841-006-9014-0

[B41] Niemelä JK Kotze DJ Venn S Penev L Stoyano I Spence J Hartley D Montes E (2002) Carabid beetle assemblages (Coleoptera, Carabidae across urban-rural gradients: an international comparison. Landscape Ecology 17: 387-401. 10.1023/A:1021270121630

[B42] Niemelä JK Kotze J Ashworth A Brandmayr P Desender K New T Penev L Samways M Spence JR (2000) The search for common anthropogenic impacts on biodiversity global network. Journal of Insect Conservation 4: 3-9. 10.1023/A:1009655127440

[B43] Palmer M (2007) Ordination Methods for Ecologists. Stillwater, Oklahoma, Oklahoma State University, Botany Department. [available from: http://ordination.okstate.edu/index.html]

[B44] Paquin P (2008) Carabid beetle (Coleoptera: Carabidae) diversity in the black spruce succession of eastern Canada. Biological Conservation 141: 261-275. 10.1016/j.biocon.2007.10.001

[B45] Peet RK Christensen NL (1980) Succession: a population process. Vegetation 43: 131-140. 10.1007/BF00121025

[B46] Peet RK Christensen NL (1987) Tree Death: Cause and Consequence. Bioscience 37: 586-595. 10.2307/1310669

[B47] Rainio J Niemelä JK (2003) Ground beetles (Coleoptera: Carabidae) as bioindicators. Biodiversity and Conservation 12: 487-506. 10.1023/A:1022412617568

[B48] Riley KN (2010) Ground beetles as biodiversity indicators for age structure in Piedmont forests? MS thesis, Winston-Salem, North Carolina: Wake Forest University.

[B49] Shannon CE Weaver W (1949) The Mathematical Theory of Communication, University of Illinois Press, Urbana, Illinois.

[B50] Simpson EH (1949) Measurement of diversity. Nature 163; 688–688. 10.1038/163688a0.

[B51] Smith TM Smith RL (2006) Community Ecology. In: Wilber B, Anderson A, Lally-Graves N (Eds) Elements of Ecology (6^th^ Edition), Pearson Education Inc., publishing as Benjamin Cummings, San Francisco, California, 6.16 – 6.19.

[B52] Sokal RR Rohlf FJ (1995) Assumptions of analysis of variance. In: Biometry: the Principles and Practice of Statistics in Biological Research. 3^rd^ Edition, Freeman and Company, New York, 397–403.

[B53] Spence JR Langor DW Niemelä JK Cárcamo HA Currie CR (1996) Northern forestry and carabids: the case for concern about old-growth species. Annules Zoologici Fennici 33: 173-184.

[B54] Thiele H-U (1977) Carabid Beetles in their Environments. Springer-Verlag, New York, 369 pp.

